# Bio-Stimulant for Improving *Simmondsia chinensis* Secondary Metabolite Production, as Well as Antimicrobial Activity and Wound Healing Abilities

**DOI:** 10.3390/plants12183311

**Published:** 2023-09-19

**Authors:** Fadia El Sherif, Munirah AlDayel, Mohammad Bani Ismail, Hind Salih Alrajeh, Nancy S. Younis, Salah Khattab

**Affiliations:** 1 Department of Biological Sciences, College of Science, King Faisal University, Al-Ahsa 31982, Saudi Arabia; felsherif@kfu.edu.sa (F.E.S.); maldayel@kfu.edu.sa (M.A.); skhattab@kfu.edu.sa (S.K.); 2 Department of Horticulture, Faculty of Agriculture, Suez Canal University, Ismalia 41522, Egypt; 3 Department of Basic Medical Sciences, School of Medicine, Aqaba Medical Sciences University, Aqaba 77110, Jordan; mohammad.ismaeil@amsu.edu.jo; 4 Department of Pharmaceutical Sciences, College of Clinical Pharmacy, King Faisal University, Al-Ahsa 31982, Saudi Arabia; 5 Zagazig University Hospitals, Zagazig University, Zagazig 44519, Egypt

**Keywords:** jojoba, angiogenesis, anti-inflammation, *Staphylococcus aureus*, *Escherichia coli*

## Abstract

*Simmondsia chinensis* is a dioecious, long-lived perennial shrub. Its leaves contain several antioxidant flavonoids that have numerous pharmacological effects. Various strategies have been explored to propagate jojoba with enhanced pharmacological values. This research evaluates the bio-stimulatory impacts of He–Ne laser seed irradiation on seed germination, plantlet growth, and alteration of the composition and bioactivities of phytochemicals in jojoba plants. Jojoba seeds were irradiated for 5, 10, and 15 min before in vitro germination. Germination, growth, and multiplication parameters were recorded during germination, multiple-shoot induction, and rooting stages. The wound healing and antimicrobial activities of methanolic extracts from plant lines obtained from the non-irradiated (control) and 10 min irradiated seeds were compared by excision wound model in Wistar male rats and zone of inhibition assay. Our study revealed that laser irradiation increased seed germination, with the highest percentage observed in seeds irradiated for 10 min. Plant lines from the 10 min irradiated seeds produced more explants with higher explant heights and numbers of leaves, more roots, and higher photosynthetic pigment contents than those of control and other laser testings. By comparing plant extracts from the control and 10 min treatments, we observed that extracts from the 10 min treatment exhibited higher percentages of wound contraction and shorter epithelialization periods. In addition, these extracts also resulted in higher levels of angiogenesis elements (VEGF, TGF-β1, and HIF-1α) and reduced the inflammation regulators (IL-1β, IL-6, TNF-α, and NFκB) in the experimental rats. In concordance, extracts from the 10 min treatment also explained raised antibacterial activities towards Staphylococcus aureus and *Escherichia coli*. Our findings show that pre-sowing seed treatment with a He–Ne laser (632.8 nm) could be a good technique for stimulating *S. chinensis* plant growth and increasing the impact compound levels and biological activities.

## 1. Introduction

*Simmondsia chinensis* L. (jojoba), a dioecious, long-lived perennial shrub in the Simmondsiaceae family, is commonly planted in semi-arid areas. [1]. The jojoba seed yields high-quality oil with medicinal and industrial uses [2]. Jojoba leaves contain numerous antioxidant flavonoid molecules that exhibit numerous pharmacological actions [3]. Ecological studies have shown that the plant variety holds significant potential in combating the process of desertification and degradation of land in arid and semi-dry regions [4]. In recent years, jojoba agricultural production has been gaining popularity and has shown promising results regarding cultivation requirements, planting densities, management strategies, productivity, propagation techniques, and genetic enhancement. Commercial jojoba plantations are developed by rooted cutting or grafting, possibly resulting in small genetic variation [1]. Seed propagation is an important source for improving plant quality and yield in plant breeding research, particularly in cross-pollinated species like the jojoba plant, where novel gene combinations are formed due to cross-pollination, resulting in offspring that differ from their parents in both genotype and phenotype [5,6]. Research indicated that in vitro derived jojoba plants develop faster than their seedling and rooted cutting counterparts and thus become significantly bigger after the first year [7].

Pre-sowing seed irradiation using a red laser at 632 nm wavelength produced by a helium–neon [He–Ne] gas laser has been proven to boost seed germination speed, seedlings and plants maturation, as well as improve the overall grade and amount of product yield [8,9,10,11,12,13]. Pre-sowing laser irradiation induces DNA polymorphism in the plants that derive from it. This DNA polymorphism could be identified using ISSR markers [14,15].

There is keen attention regarding exploring novel wound healing substances from natural resources and characterizing their mode of action. The wound healing process comprises several intersecting biological processes, including hemostasis, inflammation, proliferation, and remodeling [16]. Abnormalities in any of these stages might cause delayed wound healing progression, leading to poorly restored skin tissue. Using natural alternatives to repair damaged tissues, interventions may efficiently modify the signaling boost, components, and cytokines in the extracellular matrix (ECM) and collagen deposition [17]. Therefore, plant-based products are of imperative use in traditional wound healing therapy or even as synthetic medicament substitutes. Jojoba leaves extract presented numerous pharmacological actions, such as anti-inflammatory [18], antioxidant [19], anti-fungal, and anti-microbial properties [20]. Furthermore, a dermatological clinical study showed that jojoba oil microemulsion reduces the tazarotene irritability, enhances its placement on the skin, and achieves a good prognosis in patients with psoriatic [2].

*Staphylococcus aureus* is a coagulase-positive microorganism that reproduces on the skin and only replicates in the anterior nares of 25–30% of humans. *S. aureus* is the most well-known staphylococci member due to its extensive arsenal of virulence factors, which allow it to enter the body and produce several kinds of diseases, from benign skin and underlying tissue disorders, including abscesses, to malignant and dangerous infections, including pneumonia, bacteremia, and infective endocarditis [21]. Also, pathogenic bacteria like *E. coli* are commonly found in colonizing the human intestine and causing diarrhea. Bacterial diarrhea continues to be a health issue in both industrialized and developing nations. The likelihood of developing a serious illness, sepsis, shock, septic, or multiorgan dysfunction is still overwhelmingly influenced by bacterial infection [22,23]. As natural plant products become an alternate treatment for treating antibiotic- or drug-resistant bacteria, a rising public health problem, jojoba’s antimicrobial activity attains a greater medical relevance.

*S. chinensis* seeds were treated with He–Ne laser irradiation for the duration of 5, 10, and 15 min before in vitro culture to investigate the impacts of laser pre-treatments on seed germination, multiplication, rooting, photosynthetic pigment content, genetic variability, and phytochemical ingredients of methanolic extract from the in vitro rooted plantlets. Due to the wide aesthetic usage and the skin-related properties of jojoba, this research was further altered to find out the beneficial curing properties of the methanolic extracts of *S. chinensis* using an excision wound model as well as the antimicrobial activity of jojoba leaf methanolic extract to measure the natural properties of alterations in the bioactive ingredients as an outcome of the helium–neon (He–Ne) laser irradiation pretreatment.

## 2. Results

### 2.1. The Impacts of He–Ne Laser Treatment of Pre-Sowing Seeds on Seed Germination Percentage of S. chinensis

**Figure 1:**
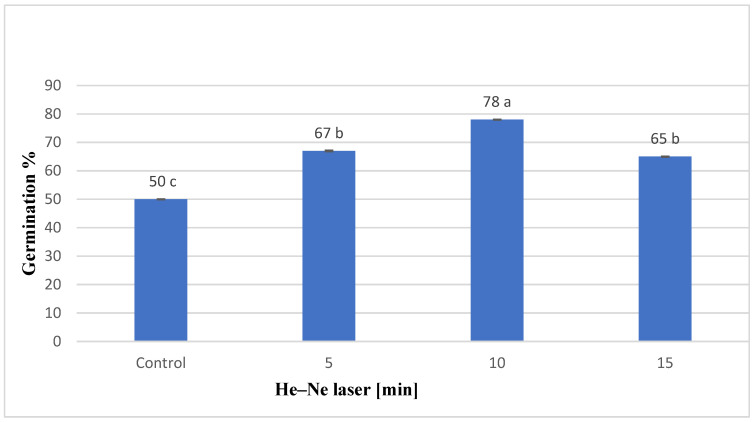
Impact of laser testing on seed germination percentage of *S. chinensis.* Means followed by the same letter above a chard are not significantly different at 0.05 level of probability according to Tukey’s test for post hoc analysis.

The data in Figure 1 showed that exposing *S. chinensis* seeds to the He–Ne laser for different periods showed a great rise in germination percentage compared to the control (non-irradiated). However, the germination percentage was lower at 15 min treatment compared to the 5 and 10 min exposure periods. Most sprouting seedlings from seeds treated with the He–Ne laser grew slowly, had abnormal shapes, and died after four weeks of germination.

### 2.2. The Impact of He–Ne Laser Pre-Sowing on In Vitro Shootlets Multiplication of S. chinensis

**Figure 2:**
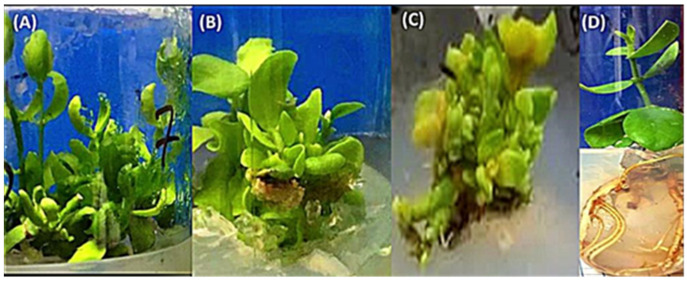
Presents the impacts of laser testing on in vitro growth of *S. chinensis*. (**A**) shows the multiplication of control plant line number 1; (**B**,**C**) are plant multiplication from laser treatments 10 and 15 min (line number 5 and 10) and (**D**) is rooted explants from 10 min laser treatment line number 8 respectively.

For the multiplication stage, 10 unique seedling lines were chosen from *S. chinensis* seeds treated for different durations by He–Ne laser and those of control (non-irradiated seeds) (Table 1). The multiplication stage was repeated three times, and the explants were transferred to new multiplication media every four weeks. Data in Table 1 and Figure 2 revealed significant differences in the number, weight, and number of leaves per shootlet produced by *S. chinensis* among different lines. When the lines were compared, line number 8 (the plant from 10 min irradiated seeds) showed the highest value of the parameters, as mentioned earlier, followed by line number 5 (the plant from 10 min irradiated seeds), and line number 2 (control, non-irradiated) had the lowest value (Table 1). Lines 4 (from 5 min irradiated seeds), 6, and 7 (from 10 min irradiated seeds), and 9 and 10 (from 15 min irradiated seeds) produced yellow leaves and weak shoots and eventually died at the end of multiplication experiments (Figure 2C).

### 2.3. The Impact of He–Ne Laser Pre-Sowing on In Vitro Shootlets Rooting and Acclimatization of S. chinensis

The rooting stage was achieved from lines 1, 2, and 3 from control (non-irradiated) and lines 5 and 8 (from 10 min irradiated seeds). The data in (Table 2) reveal that only two lines (1 and 8) of the five lines under study could produce roots after four weeks on rooting media. Line 8 (from 10 min irradiated seeds) demonstrates the greatest shoot length, weight, root number, and root length compared to the other lines (Table 2 and Figure 2D). The acclimatization procedures used in this study successfully used the previously mentioned experimental conditions. Almost 70% of the two lines’ (1 and 8) regenerated plants survived and grew vigorously.

### 2.4. The Impact of He–Ne Laser Pre-Sowing on Photosynthetic Pigment Contents of S. chinensis

Since all exposed seeds produced plantlets with higher amounts of pigments that are needed for photosynthesis than the control, it is evident that all He–Ne laser treatments favorably altered the contents of chlorophyll a (Chl a), chlorophyll b (Chl b), and carotenoid. This is supported by the results in Table 3. The line with the greatest concentrations of chl a, chl b, and carotenoids was determined to be line 8 (10 min irradiation), followed by line 5.

### 2.5. ISSR-PCR Analysis

**Figure 3:**
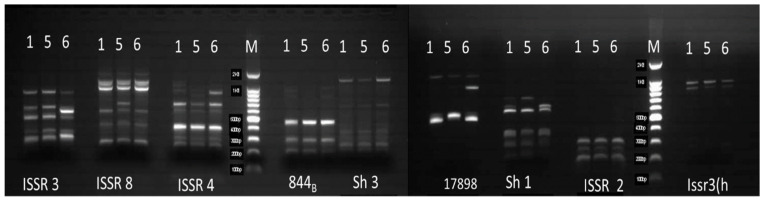
*S. chinensis* ISSR-PCR patterns with 9 primers. Lane M has the DNA marker (1 Kb DNA ladder, Takara), whereas Lanes 1, 5, and 6 include PCR products from DNA samples from the control, 10 min, and 10 min laser treatment plantlets, respectively.

Using 29 primers, DNA samples from different lines of *S. chinensis* plantlets growing in the rooting stage (control and 10 min laser irradiation) were analyzed (Table 1). Nine primers were known to develop clear amplified bands out of the thirty-one primers tested (Figure 3). The amplified amplicons were 41, with each primer amplifying two to seven fragments (Table 4). ISSR3 and ISSR8 primers produced the most amplicons (seven bands), while the ISSR3[h] primer produced the least (two bands) (Table 4). The ratio of polymorphism presented by the various primers ranged from 0% for ISSR2, 844B, and Issr3[h] primers to 57.14% for ISSR3. ISSR3, ISSR8, ISSR4, and Sh3, 17898A, and Sh1 primers were known to develop unique markers with different molecular weights (1000, 900, 800, 700, 600, 560, and 350 bp) (Table 4 and Table 5 and Figure 3). Our findings indicated DNA polymorphism between the *S. chinensis* lines under investigation.

### 2.6. Evaluation of Methanolic Leaf Product with the Help of Gas Chromatography–Mass Spectrometry (GC–MS)

Methanolic extracts of *S. chinensis* lines obtained from He–Ne laser pre-sowing treatments and the control were analyzed using GC–MS (Table 6 and Table S1). Almost 24 ingredients were present in the methanolic extracts of *S. chinensis* leaves. The name of the substances, their retention times, compositions (area percentage), molecular formulas, and molecular mass are listed in Table S1. The impact of He–Ne laser pre-sowing on the composition of selected substances such as L-proline [N-Methyl-L-proline], phenolic [1-[4-hydroxy-3-methylphenyl] ethanone], acetosyringone, and 4-hydroxyphenyl acetonitrile], cyclic secondary amine [Pyrrolidine], anhydrous sugar [levoglucosan], saccharides [methyl α-D-glucopyranoside, D-glucose and D-fructose 3-O-methyl], glycoside [arbutin], fatty acid [methyl palmitate], vitamin [l-[+]-ascorbic acid 2,6-dihexadecanoate], thiamin [L-thymidine], alkane [undecane], carboxylic acid [propanoic acid], and diterpene alcohol [phytol] were compared in Table 7. According to the data in Table 6, eight compounds were found in the extracts of all treatments; three compounds were present in the control line and 10 min He–Ne laser pre-sowing line 5, one was present in the control line and 10 min He–Ne laser pre-sowing lines 8, and four compounds were found in both lines 5 and 8 from the 10 min irradiated seeds. The levels of 1-[4-hydroxy-3-methylphenyl] methanone, methyl alpha-D-glucopyranoside, methyl palmitate, and D-fructose, 3-O-methyl were increased by about 2.1-, 3.4-, 5.4-, and 1.1-fold, respectively, in He–Ne laser pre-sowing line number 5 (Table 6). Compared to the control treatment, the He–Ne laser pre-sowing resulted in significantly lower levels of N-Methyl-L-proline, levoglucosan, D-glucose, arbutin, and acetosyringone (Table 6). Higher quantities of thymidine, undecane, propanoic acid, and phytol were created by He–Ne laser pre-sowing line number 5 (Table 6).

### 2.7. Excision Wound Healing Evaluation

#### 2.7.1. Effects of Methanolic Products of *S. chinensis* on Excised Area Shrinkage and Epithelialization

**Figure 4:**
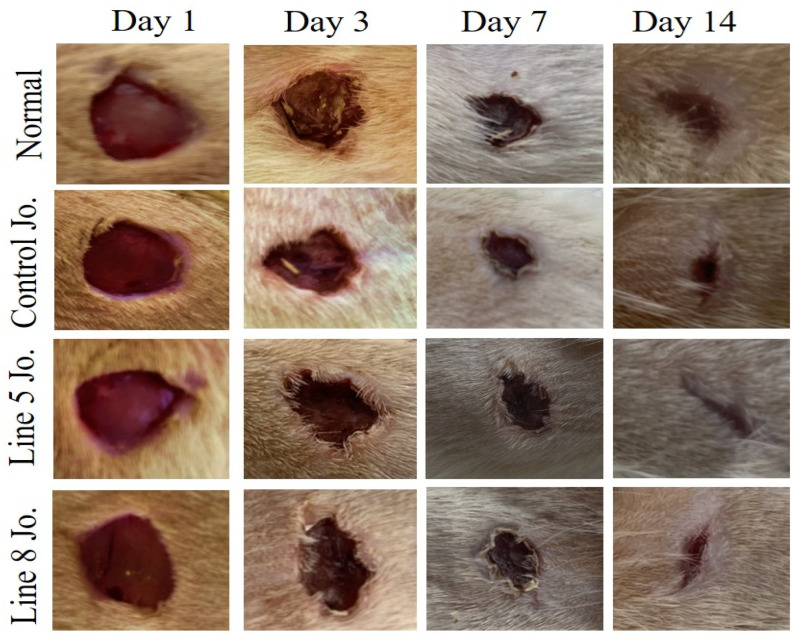
Illustrative images displaying the impact of jojoba external placement on the excision lesion remedial progression obtained from all the experimental groups.

Wound area reduction was considered for appraising the wound healing process. Demonstrative photos were taken on different days starting from the first day of treatment to assess the possibility of the methanolic extracts of *S. chinensis* leaves on the wound healing progression in the different groups (Figure 4). A bright red color was witnessed in all wounds on the first day following the wound establishment, indicating clotted blood to the underlying muscle. On the third day, visibly growing fresh skin after jojoba treatments appeared related to the normal group, reflecting the wound healing progression initiation. However, no significant difference was established among all jojoba preparations. On the 7th day, a shady brown color appeared at the site of injury in the jojoba-treated animals, signifying the crust production. In contrast, the average untreated lesions were still faintly red and swollen. On the 14th day, wounds treated with the methanolic extracts of *S. chinensis* exhibited significant wound size reduction, reaching 75.94%, 85.94%, and 80.94%, respectively, compared to the normal group (Figure 4, Table 7). Additionally, line 5 jojoba and line 10 jojoba exhibited significantly enhanced wound contraction percentage when compared to the control jojoba. On the 21st post-lesion injury day, the non-treated rats still displayed an open wound (about 10%), contrary to those treated with methanolic extracts of *S. chinensis*, which exhibited a total wound contraction (100%; Table 3). Regarding the epithelialization period, the mean period of epithelialization decreased in groups treated with the methanolic extracts of *S. chinensis*. Line 5 jojoba and Line 8 jojoba demonstrated significantly lower epithelialization periods when related to the control jojoba group. In general, He–Ne laser pre-sowing treatments not only aided in the stimulation of active substance synthesis but also resulted in the development of novel compounds that were not present in the control line.

#### 2.7.2. Effects of Methanolic Extracts of *S. chinensis* Leaves on Collagen, Hydroxyproline, and Hexosamine

**Figure 5:**
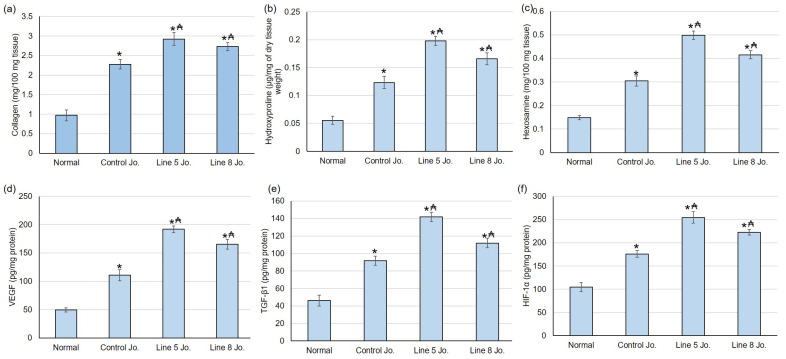
Impact of testing with jojoba on (**a**) collagen, (**b**) hydroxyproline, and (**c**) hexosamine content and on angiogenesis-related elements including (**d**) VEGF, (**e**) TGF-β1, and (**f**) HIF-1α levels in excised lesion. All data were presented as average ± SD [*n* = 6]. * specifies substantially important from a normal untreated class; ₳ specifies substantially important from control jojoba class [*p* < 0.05] by implying one-way ANOVA and Tukey’s test as a post hoc evaluation.

Collagen is an essential pack of amino acids present outside the cell in the skin. Its major constituent is hydroxyproline. Therefore, the hydroxyproline content in the tissues indicates any change in collagen formation, thereby signifying the healing phenomenon within the damaged skin. In the current study, normal rats exhibited the lowest quantities of collagen, hydroxyproline, and hexosamine, whereas all groups treated with the methanolic extracts of *S. chinensis* showed significantly higher values. Additionally, the line 5 jojoba and line 10 jojoba groups presented higher collagen, hydroxyproline, and hexosamine content when correlated to the control jojoba group, as illustrated in Figure 5a–c.

#### 2.7.3. Effects of Methanolic Extracts of *S. chinensis* Leaves on Angiogenesis Factors

Throughout the wound repair process, angiogenesis provides the essential nutrients needed and serves structural repair by forming granulation tissue. The outcomes of the existing study revealed that the angiogenesis elements, including VEGF, TGF-β1, and HIF-1α contents in the normal group, were inferior to the methanolic extracts of *S. chinensis*-treated groups. Furthermore, line 5 jojoba and line 10 jojoba groups presented significant upsurge in HIF-1α, TGF-β1, and VEGF levels related to control-jojoba-treated animals (Figure 5d–f).

#### 2.7.4. Effects of Methanolic Extracts of *S. chinensis* Leaves on the Inflammatory Mediators

**Figure 6:**
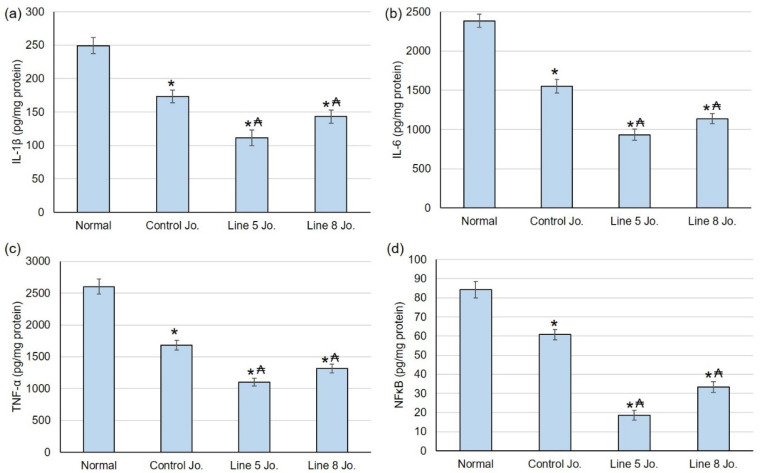
Jojoba oil medication had a consequence on (**a**) IL-1β, (**b**) IL-6, (**c**) TNF-α, and (**d**) NF-κB in resection trauma, among other markers of inflammation. All data were presented as average ± SD [*n* = 6]. * specifies substantially important from a normal untreated class; ₳ specifies substantially important from control jojoba class [*p* < 0.05] by implying one-way ANOVA and Tukey’s test as a post hoc evaluation.

Our next step was assessing the wound healing course’s inflammation degree. Untreated animals exhibited a substantial intensification in the inflammatory mediators, including IL-1β, IL-6, TNF-α, and NFκB. However, animals treated with the methanolic extracts of *S. chinensis* displayed decreased inflammation, as indicated by the lowered levels of inflammatory mediators compared to normal animals. Furthermore, the animal groups treated with line 5 jojoba and line 10 jojoba presented a significant reduction in IL-1β, IL-6, TNF-α, and NFκB when linked to controls (Figure 6).

### 2.8. The Effect of Jojoba on Antimicrobial Activity of the Extraction

The disc migration studies (Table 8) showed that different levels of antibacterial capability were present for microbial genera *S. aureus* and *E. coli* in methanolic products from the vegetative parts of the jojoba bush. The methanolic concentrate from leaves conditioned with He–Ne laser boosted its antibacterial efficacy towards *S. aureus* (almost equivalent to that displayed by 10 g of Imipenem). Still, it was not as potent against E. coli between the two examined microbe varieties. Unfiltered (control) samples were more potent regarding all infectious microbes (*S. aureus* and *E. coli*). Samples from bushes exposed to the He–Ne laser revealed lower anti-*E. Coli* efficacy than those from plants used as controls. The information in Table 9 demonstrates the antibacterial efficacy of jojoba leaf methanolic extracts on the various examined species. The data showed that *E. coli* exhibited a smaller zone of inhibition and a reduced sensitivity at all methanolic concentrations. *E. coli* was the least sensitive bacterium, inhibited at concentrations of control lines 1, 5, and 8, respectively, with 10, 12, and 14 mm inhibition zones. *S. aureus* was the most sensitive, with an 18 mm zone of inhibition at line 8 concentration extract, 14 mm at line 5, and 13 mm at the control line 1 (Table 8).

## 3. Discussion

The application of laser irradiation at a suitable wavelength has the potential to improve crop quality and quantity. The current study investigated the influence of He–Ne laser treatment at a wavelength of 632 nm conducted for different periods on pre-sowing seeds on germination percentage, multiplication, and rooting enhancers for *S. chinensis* in vitro plantlets lines established from irradiated seeds. Furthermore, the effects of irradiation testing on the chlorophyll a, chlorophyll b, and carotenoid ingredients, the phytochemical contents of methanolic extracts generated from rooted plantlets lines, and DNA polymorphism utilizing ISSR techniques were investigated.

The ability of seeds to receive and store radiant energy is critical in the bio-stimulation process. Our findings showed that He–Ne laser pre-sowing seeds considerably boosted seed germination percentage compared to the control treatment. Laser bio-stimulation has already enhanced many crops’ seed germination rates [12,13,24,25]. Line selection is a tool for producing high-quality plants. It was accomplished by choosing good-quality seedlings from *S. chinensis* plant seedlings germinated in vitro and multiplying each line independently. In the existing investigation, we chose 10 different *S. chinensis* lines from seedlings formed from germinated seeds that were treated with varied periods of He–Ne laser and based on their higher growth. The multiplication rate varied between lines. At the end of the multiplication steps, some lines failed to produce healthy explants. Line 8 (10 min He–Ne laser pre-sowing seeds) significantly enhanced the number, weight, and number of leaves per shootlet formed by *S. chinensis*. The multiplication stage plant growth increase in line 8 is accompanied by a significant increase in rooting characteristics and plant photosynthetic pigments. Laser bio-stimulation effects on plant growth were reported on *S. chinensis* [9] and eventually quoted by researchers in different plant genera [26,27]. Within the three irradiation periods tested in this study, the 10 min pre-sowing irradiation lines produced the highest growth. Negative consequences were detected when the irradiation duration exceeded 15 min [28,29].

The 10 min He–Ne laser pre-sowing seeds significantly boosted photosynthetic pigments in the rooting stage compared to control lines. The laser-induced increase in photosynthetic pigments could be attributed to its stimulatory action in chlorophyll production. The He–Ne laser has been shown to increase the concentration of photosynthetic pigments [10,24,30]. ISSR molecular markers have emerged as an intriguing method for analyzing the genetic variation within and between populations. Also, they can be employed without knowledge of genomic DNA sequence information, as in *S. chinensis* [31,32,33,34]. Using these markers, we discovered DNA variation between the *S. chinensis* lines under examination in this study. Changes in the ISSR profile generated by laser treatments in various lines can be interpreted as changes in genomic DNA template stability, and these genetic consequences can be directly compared to changes in other parameters. Several research groups have also used ISSR markers to study He–Ne-laser-induced genetic variation in plants [14,15]. Secondary metabolite biosynthesis and accumulation can be influenced by several genetic, ontogenic, morphogenetic, and environmental variables [35]. In this investigation, each *S. chinensis* line had a different percentage of bioactive compounds, and some of these compounds were not discovered in one line but were detected in others. Bioactive compounds are more abundant in plants generated from laser-irradiated grains or vegetative reproduction organs [15,36]. In addition to laser-assisted growth, there was a rise in some bioactive compounds compared to the control lines. Even with higher growth, plants from the 10 min irradiated seeds line showed lower N-Methyl-L-proline, Levoglucosan, D-Glucose, l-[+]-Ascorbic acid 2,6-dihexadecanoate, Arbutin, and Acetosyringone contents as compared with the control line. These variations would be regarded as genetic variation within the *S. chinensis* line as a result of the He–Ne laser pre-sowing seeds utilized.

Wounds treated with any of the methanolic extracts of *S. chinensis* exhibited a higher wound reduction percentage and lowered period of epithelialization compared to animals treated with normal. Meanwhile, line 5 jojoba and line 10 jojoba demonstrated significantly enhanced wound contraction percentage and lowered epithelialization time compared to the non-irradiated control extract. An earlier study executed by [37] explored the lesion-curing characteristics of jojoba liquid wax (JLW) in vitro on HaCaT keratinocytes and human dermal fibroblasts that participated in the injured skin regrowth. The study revealed that JLW enhanced the lesion regrow capacity of fibroblasts and keratinocytes by activating the PI3K/Akt/mTOR pathway, an important constituent of the average lesion-curing procedure.

Additionally, in the current study, normal untreated animals exhibited the lowest collagen, hydroxyproline, and hexosamine contents. In contrast, the groups treated with the methanolic extracts of *S. chinensis* demonstrated higher collagen, hydroxyproline, and hexosamine contents. Furthermore, line 5 jojoba and line 10 jojoba groups presented further high contents of collagen, hydroxyproline, and hexosamine, unlike the non-irradiated control jojoba group. Collagen, an essential constituent of the ECM, plays a crucial part in regulating the lesion-healing phases [38]. Hydroxyproline is an amino acid incorporated in collagen synthesis. Therefore, hydroxyproline concentration measures collagen synthesis within the healing tissue [39]. Greater hydroxyproline content indicates an increased rate of wound healing. Biochemical investigation in the current study showed amplified hydroxyproline content, reflecting an augmented cellular proliferation and improved collagen synthesis. Additionally, amplified hexosamine content indicates the maintenance of collagen molecules by improving electrostatic and ionic interactions.

The proliferative duration of lesion curing is manifested by the existence and abundance of diverse factors, including angiogenesis elements. The secreted factors include VEGF, TGF-β, HIF-1α, and matrix metalloproteases (MMPs). HIF-1α, TGF-β1, and VEGF are involved in the healing course by inspiring cell proliferation by turning on numerous practices, including expanding the extracellular matrix (ECM), re-epithelialization, and angiogenesis [40]. The angiogenesis elements, including VEGF, TGF-β1, and HIF-1α contents in the normal group, were inferior to methanolic extracts of *S. chinensis*-treated groups. Furthermore, line 5 jojoba and line 10 jojoba groups presented a substantial escalation in HIF-1α, TGF-β1, and VEGF levels when related with control-jojoba-treated animals, indicating an augmented re-epithelialization and angiogenesis process.

Regarding inflammation, earlier studies showed elevated inflammatory mediators during wound healing. When the vascular wall is disrupted, chemotaxis promotes the relocation of inflammatory cells and initiates consecutive infiltration of neutrophils, macrophages, and lymphocytes within the wound [41,42]. In the current study, normal animals revealed a significant intensification in the inflammatory regulators IL-1β, IL-6, TNF-α, and NFκB, whereas rats treated with the methanolic extracts of *S. chinensis* displayed decreased inflammation, as justified by the considerably lesser stage of inflammatory regulators when related to untreated animals. Furthermore, the animal groups treated with line 5 jojoba and line 10 jojoba presented a significant decrease in IL-1β, IL-6, TNF-α, and NFκB when linked to control jojoba. An earlier study showed that jojoba oil reduced carrageenin-induced rat paw edema, croton-oil-induced ear oedema, and lipopolysaccharide (LPS)-induced air pouch inflammation, signifying the potent anti-inflammatory efficacy of jojoba [18]. Due to the rise in antibiotic-resistant bacteria, there is an increased need for new antimicrobial agents.

Several research teams have proposed that jojoba might have these qualities. Additionally, Abu-Salem and Ibrahim have demonstrated the remarkable efficacy of plant latex and root extract against bacteria and fungus. Separate studies have discovered that jojoba oil has antibacterial and antifungal properties [43]. Therefore, it would appear that the active agent(s) are not confined to a particular area of the plant. It is crucial to note, nevertheless, that numerous other reports evaluating analogous extracts failed to detect any antibacterial action. The differences in the cultivation and genetic makeup of the plants and the bacterial strains used could all contribute to this variance. Further, [3,18,19,23,44] used the presence or absence of inhibition zones to qualitatively evaluate the antimicrobial activity of several jojoba leaves’ extracts against the used bacteria. The results demonstrated differences between the antibacterial activity of the three jojoba leaf extract lines against the tested microbial species. It was demonstrated that the jojoba plant can be used as a source of bioactive molecules, and this direction should also be developed. For instance, Arbutin is a skin depigmenting agent with antimelanogenic and antioxidant properties [45]. Another molecule is methyl palmitate, which attenuated lipopolysaccharide-induced acute lung injury [46], reversed estradiol-benzoate-induced endometrial hyperplasia [47], and ameliorated ethanol-induced gastric mucosal injury via targeting MAPKs, NF-κB, and PI3K/AKT pathways [48]. Also, aromatic aldehydes like o-vanillin (2-Hydroxy-3-methoxybenzaldehyde) reduced the complications of sickle cell disease (SCD) by interaction with sickle cell hemoglobin (HbS) and reduced polymerization and RBC sickling in sickle cell patients [49]. Oleic acid (OA) is an effective biomolecule as it may directly regulate both the synthesis and activities of antioxidant enzymes as well as inhibit the pro-inflammatory cytokines and activate the anti-inflammatory ones [50]. Additionally, numerous studies have reported an inhibition in cell proliferation induced by OA in different tumor cell lines. OA suppressed the over-expression of HER2, a well-characterized oncogene [51]. Also, diets rich in oleic acid exhibited beneficial effects in inflammatory-related diseases [52]. Another bioactive molecule is propanoic acid, which is known to have antimicrobial and immune-modulatory properties and is generally recognized as a safe (GRAS) food ingredient by the FDA, where it acts as an antimicrobial agent for food preservation and a flavoring agent [53]. Furthermore, phytol, a chlorophyll component, produces antihyperalgesic, anti-inflammatory, and antiarthritic effects through the NFκB pathway [54].

## 4. Materials and Methods

### 4.1. Helium–Neon Laser Irradiation

Seeds of *S. chinensis* were bought from the Department of Medicinal and Aromatic Plants (ARC), Institute for Horticultural Science, Giza, Egypt. Jojoba seeds (average weight 0.86 to 0.85 g) were segregated into three laser treatment groups. The seeds in the ‘control’ group were not exposed to radiation, and the remaining groups were exposed to laser for five, ten, and fifteen minutes, respectively. A LeyboldR 471,830 device delivering a linearly polarized He–Ne laser having a wavelength of 632 nm and a power density of 5.5 mW/mm^2^ (Department of Physics, College of Science, King Faisal University) was used. The seeds were stored in plates 40 mm below the laser instrument.

### 4.2. Impacts of Laser Treatments on S. chinensis Seed Growth

To disinfect, seeds were wetted in running water for 10 min before soaking them in 0.5 g/L Carbomar fungicide (Anmar for Development Agriculture Trading) for 15 min. Afterward, seeds were surface-sterilized with 70 percent ethanol solution for 30 s, then soaked in 0.1% aqueous mercuric chloride (HgCl_2_) solution for 5 min before being washed triple with sterile tap water under laminar airflow. The sterilized grains were germinated on Murashige and Skoog (Caison Labs, Smithfield, UT, USA) medium mixed with 30 g/L sucrose and 6 g/L agar (Industrias R O K O, S.A, Llanera, Asturias, Spain). A completely randomized experimental design with 50 repetitions was used. Further, 50 seeds were grown in 50 mL tubes filled with 10 mL half MS medium, one seed per 50 mL tube. A growth chamber with 24 ± 2 °C and 16 h photoperiods provided by an illuminated 4000 Lux light intensity (Phillips TLM 40 W/33RS) was utilized to grow plants. The seed germination percentage of each laser treatment was recorded after 4 weeks. The in vitro germinated plantlets were employed in all subsequent propagation investigations.

### 4.3. Effect of Laser Treatments on S. chinensis Shoot Multiplication

The 0.5–1 cm tall, one-month-old stem cuttings from 10 distinct laser *S. chinensis* lines (showing similar growth potential as seedlings) were grown on MS medium mixed with 30 g/L sucrose, 6 g/L agar, and 1.0 mg/L 6-benzyl aminopurine (BAP, Bio Basic INC^®^, Markham, ON, Canada) [9]. Cultures were grown in 40 mL culture jars. Subculture was achieved by placing stem-cutting portions into the fresh multiplication media every four weeks. After 12 weeks, the following data were collected: explant fresh weight (g), length of the highest explant (cm), and shoots and leaves number per explant [n].

### 4.4. Effect of Laser Treatments on S. chinensis Root Induction

A total of 10 *S. chinensis* lines were chosen for the stage of root growth. Shoot tips (0.5–1 cm in height) were replaced on half MS medium with 1.0 mg/L Indole-3-Butyric (IBA, Bio Basic INC^®^, Canada), and 10 culture replicates (50 mL tubes) were prepared for each line. The explant height (cm), fresh weight (g), number of leaves/explant [n], number of roots/explant, and highest root length were all measured after one month of culture. In vitro rooted seedlings of five months of age were cleaned, soaked in 1% rizolex^®^ fungicide for 10 min, and transferred to plastic pots filled with wet sand in a greenhouse. The survival ratio of ex vitro acclimatized plantlet was determined two months later.

### 4.5. Determination of Photosynthetic Pigment Contents

The quantities of chlorophyll a, b, and carotenoid were quantified in explant leaves from the rooting stage using 80% acetone as a solvent, as reported by [55].

### 4.6. GC–MS Evaluation

At the rooting stage, plantlet leaves from various *S. chinensis* lines were air-dried at room temperature. Further, 1 g of homogenized air-dried leaves from three randomly selected plantlets was included in a 28 mL marked cultivated test tube and defatted for one day in 30 mL of methanol [96%] while mixed at 100 rpm on a rotary mixer. The products were filtered via a 0.2 µm syringe filter before being entered in the GC–MS system in a volume of 22 µL. The GC–MS evaluation was carried out by the Department of Chemistry, College of Science, King Faisal University, Kingdom of Saudi Arabia, according to [56].

### 4.7. Genomic DNA Separation and ISSR-PCR Evaluation

The genomic DNA was obtained from the leaves of three randomly selected six-month-old *S. chinensis* plantlets at the rooting stage using the Qiagen-DNA mini kit (cat. No. 51306). ISSR-PCR test was carried out using a pool of genomic DNAs from each line group’s three plantlets, as described by [57]**. Table 9 shows the names and combinations of the ISSR-PCR primers.**

**Table 9:** Primer identities and combinations for ISSR-PCR testing.

Primer Code	Combination	Primer Code	Sequence
ISSR 1	[AG]_8_YC	HB_11_	[GT]_6_CC
ISSR 2	[AG]_8_YG	HB_12_	[CAC]_3_GC
ISSR 3	[AC]_8_YT	HB_13_	[GAG]_3_GC
ISSR 4	[Ac]_8_YG	Sh 1	[AG]_8_CTC
ISSR 5	[GT]_8_YG	Sh 2	[AG]_8_CTG
ISSR 6	CGC[GATA]_4_	Sh 3	[AC]_8_CTT
ISSR 7	GAC[GATA]_4_	Sh 4	[AC]_8_CTG
ISSR 8	[AGAC]_4_GC	Sh 5	[GT]_8_CTG
ISSR 9	[GATA]_4_GC	Issr2[h]	CAC[TCC]_5_
ISSR 10	[GACA]_4_AT	Sh 8[h]	[AGAC]_4_GC
814_A_	[CT]_8_TG	Sh 9[h]	[GATA]_4_GC
844_A_	[CT]_8_AC	Issr3[h]	TTT[TCC]_5_
844_B_	[CT]_8_GC	Issr10[h]	[TCC]_5_NAC
17898_A_	[CA]_6_AC		
17899_A_	[CA]_6_AG		
HB_9_	[GT]_6_GG		

### 4.8. In Vivo Wound Healing Experiment

#### 4.8.1. Animal’s Acquisition and Ethical Agreement

To assess the wound healing ability of *S. chinensis*, a full-thickness excisional wound model was executed. Wistar male rats (200–220 g) were purchased from King Saud University, Riyadh, Kingdom of Saudi Arabia. At King Faisal University, all the investigations followed the “Ethical Conduct for the Use of Animals in Research” Regulations. The King Faisal University Animal Research Ethics Board approved all veterinary management and examination procedures with an ethical consent code (KFU-REC-2023-FEB-ETHICS564).

#### 4.8.2. Excisional Wound

For anesthesia, ketamine (100 mg/kg) and xylazine (10 mg/kg) were injected intraperitoneally. Rats’ thoracic fur was clipped using a powered razor, and areas of injury were externally sterilized and cleaned with 70% ethanol. Next, a scalpel blade was used to remove the entirety of an excised lesion on the rear epidermis of rats near the dorsal collar aspect that was 2 cm wide (circumferential area: 4 cm^2^). After the operation, the rats were placed individually in metabolic cages.

#### 4.8.3. Experimental Design

Wound-operated rats were haphazardly divided into four groups [*n* = 6]. The first one, where the animals were treated with ointment base, was considered a normal untreated group. In the control jojoba group, the animals have been treated with control jojoba solution (2% *w*/*w*). The third and fourth groups in which animals were treated with jojoba lines 5 and 10 were called line 5 jojoba and line 10 jojoba, respectively.

#### 4.8.4. Preparation of Jojoba for Topical Application

Different jojoba preparations were prepared in Vaseline 2% (*w*/*w*) to formulate a simple ointment. Jojoba ointments were applied topically on the wound once daily throughout the experiment, as mentioned before [58]. Prepared jojoba ointments were applied directly to the open wound injury until the skin was restored. No antibiotic was administered, and the rats were observed daily for infection. A digital camera (Canon Inc., Tokyo, Japan) was utilized to record the reduction in the wound area size.

#### 4.8.5. Wound Contraction % and Epithelialization Time

The extent of the wound decrease rate (%) is the proportion of lesions that do not progress past their first stage. The injured area was drawn on a translucent drawing document, and the wound’s outermost area was estimated. As previously mentioned, analyses of the wound closure % were completed on days 3, 7, 14, and 21 [59]. Wound region on day 0—the region of the wound on the day [n]/Wound region on day 0] × 100 = proportion of wound healing (n is the number of days). Epithelialization is a crucial element of the wound restorative process and is regularly used as a defining factor of effective wound closure [60]. After the procedure, specimens of the injured regions were removed, homogenized, and whirled at 5000× *g* for 10 min. The amounts of hydroxyproline, collagen, hexosamine, angiogenesis-related factors, and cytokines associated with inflammation were measured in the sediment.

#### 4.8.6. Assessment of Jojoba on Hydroxyproline, Collagen, and Hexosamine

Hydroxyproline content within the lesion region was determined as described earlier [61]. The hydroxyproline content was measured using the standard linear curve and shown as µg/mg of dried mass of the tissue. The injured tissue samples were stripped of fat in chloroform: methanol [2:1 *v*/*v*] and evaporated in acetone to determine the collagen and hexosamine concentrations. Weighed tissues were hydrolyzed in 6 N HCl for 18 h at 110 °C, evaporated, and then reconstituted with an estimated amount of water as previously reported [62].

#### 4.8.7. Assessment of Jojoba on the Angiogenesis Factors

Using ELISA kits acquired from LifeSpan BioSciences, Seattle, WA, USA, Vascular endothelial growth factor [VEGF; LS-F5482], transforming growth factor β1 [TGF-β1; LS-F24972], and hypoxia-inducible factor 1-alpha [HIF-1α; LS-F4225] levels were all assessed in the wound tissue homogenates.

#### 4.8.8. Assessment of Jojoba on the Inflammatory Cytokine

ELISA kits that were acquired from LifeSpan BioSciences and used according to the manufacturer’s guidelines to measure tumor necrosis factor-alpha (TNF-α; LS-F23150), interleukin 8 (IL-8; LS-F9753), and interleukin 1β levels.

### 4.9. Determination of Jojoba Methanolic Leaves Extract Antimicrobial Property by Disc Diffusion Assay

The methanolic extracts of jojoba leaves were developed as described in Section 2.7. The King Faisal University College of Medicine provided the *S. aureus* and *E. coli* (ATCC 8739) bacterial strains. The disc diffusion assay was carried out by [63], and all the microbial species were cultured in nutritious agar (Sigma Aldrich, St. Louis, MI, USA, Cat. No. 7014) [64]. Further, 100 µL of an overnight microbial culture was included in 10 mL of nutrient broth as an inoculum. The cultures were cultivated at 37 °C at 200 rpm until their turbidity reached 0.3 at 600. Additionally, 5 mL of the homogeneous colony of each bacterial strain was placed to separate food-grade agar dishes to ensure the colony was disseminated evenly on the gelatin. Each variant of bacteria was put onto twelve surfaces of agar. Before applying the diagnostic mixture plates to the agar, all infected plates had to be stored in the quarantine cabinet with the covers off for the additional fluid to absorb. On the agar surface of each immunization dish, a set of six 3 mm radius discs holding different experiment solutions were deposited. The jojoba sample solutions from the control line 1, line 5, and line 8 methanolic leaf extracts were placed on four discs.

On the other hand, the opposite side of the disc had a DMSO: water [v:v 1:1] solution. The 10 g imipenem therapeutic cartridge (cat. no. 7052) was acquired from Condalab in Madrid, Spain. The dimensions of the area of suppression were then determined after every dish underwent incubation at 37 °C for an extended period of time.

### 4.10. Statistical Analysis

The average and standard deviation (SD) of the data are presented. One-way ANO-VA was used for contrasting multiple comparisons, and the sensitivity threshold for Tukey’s test for post hoc analysis was set at 0.05 [64].

## 5. Conclusions

*S. chinensis* grows optimally in vitro after irradiating the pre-sowing seed with He–Ne laser at 632 nm. The 10 min seed irradiation produced lines with the greatest number of shoots and roots and the highest content of photosynthetic pigments. In contrast, most sprouting seedlings exposed to the 5 and 15 min He–Ne laser grew slowly, had abnormal shapes, and died after four weeks of germination. Significant changes were noticed in phytochemical composition in the leaves of different lines at the roots stage in laser-pre-treated plants and controls. Genetic variance was observed in explants extracted from laser-treated seeds compared to control plants using ISSR analysis with nine primers. Methanolic extracts of *S. chinensis* exhibited enhanced wound healing properties via augmenting angiogenesis and suppressing inflammation. Our findings show that pre-sowing seed treatment with a He–Ne laser (632.8 nm) could be an effective technique for stimulating *S. chinensis* plant growth and increasing the impact compound contents and biological activities. More research is needed to understand the effect of the He–Ne laser (632.8 nm) on *S. chinensis* plant growth.

## Figures and Tables

**Table 1:** Effects of laser treatment on multiple-shoot induction of *S. chinensis* lines.

Laser Treatments [min]	*S. chinensis* Lines	No. of Shoots/ Explant [n]	Explant’s Fresh Weight [g]	No. of Leaves/Explant [n]
Control	1	20.2 c *	3.61 g	54.25 d
Control	2	8.1 i	3.9 f	23.05 h
Control	3	18.3 e	1.75 j	25.20 g
5	4	19.2 d	3.2 h	35.25 f
10	5	21.2 b	8. 01 b	82.15 b
10	6	12.05 g	5.35 c	55.35 c
10	7	12.20 g	4.9 e	42.15 e
10	8	28.2 a	8.65 a	105.15 a
15	9	10.3 h	2.15 i	25.3 g
15	10	15.2 f	5.15 d	13.1 i

* Means followed by the same letter within a column are not significantly different at 0.05 level of probability according to Tukey’s test for post hoc analysis.

**Table 2:** Impacts of laser testing on root induction of *S. chinensis* lines.

Laser Treatments [min]	*S. chinensis* Lines	Shoot Length [cm]	Explant’s Fresh Weight [g]	No. of Leaves/Explant [n]	Length of the Longest Root [cm]	No. of Roots/ Explant [n]
Control	1	3.88 b *	0.425 b	6.5 c	1.25 b	0.75 b
Control	2	3.00 b	0.45 ab	18.0 a	0.00 c	0.00 c
Control	3	3.25 b	0.70 ab	13.0 ab	0.00 c	0.00 c
10	5	5.30 a	0.52 ab	6.7 bc	0.00 c	0.00 c
10	8	5.50 a	1.00 a	7.3 bc	4.50 a	

* Means followed by the same letter within a column are not significantly different at 0.05 level of probability according to Tukey’s test for post hoc analysis.

**Table 3:** Impacts of laser testing on chlorophyll a, b, and carotenoids ingredients of *S. chinensis* lines.

Laser Treatments [min]	*S. chinensis* Lines	Chl a [mg/100 g F.W.]	Chl b [mg/100 g FW.]	Carotenoids [mg/100 g FW.]
Control	1	61.09 c *	18.85 c	61.424 b
Control	2	55.40 c	16.04 c	56.94 b
Control	3	69.30 c	20.20 c	70.80 b
10	5	109.39 b	32.47 b	111.46 a
10	8	131.31 a	38.44 a	118.75 a

* Means followed by the same letter within a column are not significantly different at 0.05 level of probability according to Tukey’s test for post hoc analysis.

**Table 4:** Following ISSR-PCR testing with 9 initiators, four *S. chinensis* line seedlings raised in planting substance using different laser procedures were examined for the total amount of augmented pieces (AF), monomorphic stripes (MB), and polymorphics.

Laser Treatments [min]	*S. chinensis* Lines		Total	Primers								
				ISSR 2	ISSR 3	ISSR 4	ISSR 8	844B	17898A	Sh 1	Sh 3	Issr3 [h]
		AF	41	3	7	6	7	3	4	5	4	2
		MB	29	3	3	5	6	3	2	2	3	2
		PB	12	0	4	1	1	0	2	3	1	0
		PB%	29.27	0	57.14	16.67	14.29	0	50	60	25	0
		SM	8	0	2	1	1	0	2	1	1	0
Control	1	AF	33	3	4	5	6	3	3	4	3	2
		SM	1	0	0	0	0	0	1	0	0	0
10	5	AF	34	3	5	5	7	3	2	4	3	2
		SM	2	0	0	0	1	0	0	1	0	0
10	8	AF	36	3	6	6	6	3	3	3	4	2
		SM	5	0	2	1	0	0	1	0	1	0

**Table 5:** Using specimens of DNA from *S. chinensis* seedlings stimulated by different lasers, nine promoters were used to generate distinct indicators, with the approximate molecular weight [bp] of each indicator.

Laser Treatments [min]	*S. chinensis* Lines	ISSR Primers					
		ISSR3	ISSR8	ISSR4	Sh3	17898A	Sh1
Control	1					1000	
10	5		900				800
10	8	600 350		1000	700	560	

**Table 6:** Comparison of selected secondary metabolites from various *S.immondsia chinesnsis* lines as identified by GC–MS in methanolic leaf extracts from the corresponding plantlets at the rooting stage. Each area [%] was calculated from the measurements obtained from the methanolic extracts of 3 plantlets.

Phytochemical	Composition [Area %]		
	*S. chinensis* Lines		
	Control [1]	5	8
N-Methyl-L-proline	14.46 a *	5.47 b	0.61 c
1-[4-hydroxy-3-methylphenyl]ethanone	0.63 c	1.35 a	1.17 b
Pyrrolidine	0.08 c	0.15 b	0.49 a
Methyl alpha-D-glucopyranoside	0.29 c	11.82 a	4.48 b
Levoglucosan	3.86 a	2.12 b	0.24 c
D-Glucose	2.92 a	0.59 b	0.19 c
Methyl palmitate	0.21 b	1.14 a	0.20 b
l-[+]-Ascorbic acid 2,6-dihexadecanoate	0.60 a	0.58 a	0.50 a
D-Fructose, 3-O-methyl	60.15 b	68.53 a	0.00 c
Arbutin	0.30 a	0.06 b	0.00 c
Acetosyringone	0.21 a	0.09 b	0.00 c
[4-hydroxyphenyl] acetonitrile	0.75 a	0.00 b	0.76 a
L-Thymidine.	0.00 c	0.34 a	0.18 b
Undecane	0.00 c	1.02 a	0.14 b
Propanoic acid	0.00 c	0.69 a	0.49 b
Phytol	0.00 c	0.40 a	0.17 b

* Means followed by the same letter within a column are not significantly different at 0.05 level of probability according to Tukey’s test for post hoc analysis.

**Table 7:** Wound contraction % and epithelialization period in the various examination classes.

Groups	Lesion Shrinkage %				Epithelialization Period [Days]
	3rd Day	7th Day	14th Day	21st Day	
Normal	12.33 ± 1.4	30.94 ± 2.04	64.96 ± 3.1	90.6 ± 4.45	22.05 ± 1.42
Control Jojoba	16.95 ± 2.11 *	50.71 ± 2.2 *	75.94 ± 2.17 *	100	18.5 ± 0.5 *
Line 5 Jojoba	17.75 ± 1.91 *	60.76 ± 1.87 * ₳	85.94 ± 3.48 * ₳	100	16.0 ± 0.61 * ₳
Line 8 Jojoba	15.96 ± 1.51 *	55.83 ± 1.2 * ₳	80.94 ± 2.92 * ₳	100	16.0 ± 1.02 * ₳

All data were presented as average ± SD [*n* = 6]. * specifies substantially important from a normal untreated class; ₳ specifies substantially important from control jojoba class [*p* < 0.05] by implying one-way ANOVA and Tukey’s test as a post hoc evaluation.

**Table 8:** Area of resistance (mm) displayed by multiple experiment mixtures on dishes treated individually with Escherichia coli ATCC8739 and Staphylococcus aureus. The average of three duplicate dishes was used to obtain every average value imipenem 10.

Test Solution	Inhibition Zone [mm]	
	*S. aureus*	*E. coli*
Jojoba (control)	13 ± 1 d *	10 ± 2 d
Jojoba extract line 5	14 ± 1 c	12 ± 0 c
Jojoba extract line 8	18 ± 2 ab	14 ± 0 b
Imipenem 10 µg	18 ± 3 a	15 ± 2 a

* Means followed by the same letter within a column are not significantly different at 0.05 level of probability according to Tukey’s test for post hoc analysis.

## Supplementary Materials

The following supporting information can be downloaded at: https://www.mdpi.com/article/10.3390/plants12183311/s1, Table S1: Components identified by GC–MS analysis in the *S. chinensis* Lines methanolic leave extracts from different line groups. Each area [%] was calculated from the measurements obtained from the methanolic extracts of 3 plantlets from [rooting stage].
