# Supplementary material for: Bio-Stimulant for Improving Simmondsia chinensis Secondary Metabolite Production, as Well as Antimicrobial Activity and Wound Healing Abilities
Source: Plants (Basel). 2023 Sep 19;12(18):3311. doi: 10.3390/plants12183311 (PMC10536608; doi:10.3390/plants12183311)
Supplement: Supplementary file 1 [file plants-12-03311-s001.zip › plants-2599386-supplementary.pdf]

Supplementary Table S1. Components identified by GC–MS analysis in the *S. chinensis* Lines methanolic leave extracts from different line groups. Each area [%] was calculated from the measurements obtained from the methanolic extracts of 3 plantlets from [rooting stage].

Laser treatments [min]	*S. chinensis* Lines	Peak	Essential Oil Compounds	RT, min	Area, %	Molecular Weight	Molecular Formula
Control	1	1	4-Methyl-1,3-dioxane	10.71	0.29	102.13	C5H10O2
		2	N-Methyl-L-proline	11.636	14.46	129.16	C6H11NO2
		3	1-methylpyrrolidine-2-carboxylic acid	11.758	7.53	129.16	C6H11NO2
		4	[R]-Dimethyl 2-hydroxysuccinate	13.338	0.09	162.14	C6H10O5
		5	hexahydro-1,3-diazocine-2[1H]-thione	14.149	0.08	144.24	C6H12N2S
		6	2,6-Diethylcyclohexanone	14.211	0.06	154.25	C10H18O
		7	1-[4-hydroxy-3-methylphenyl]ethanone	14.734	0.63	150.17	C9H10O2
		8	Pyrrolidine	16.206	0.08	71.12	C4H9N
		9	Methyl alpha-D-glucopyranoside	17.34	3.9	194.18	C7H14O6
		10	2-Methoxyphenylacetonitrile	17.674	0.21	147.17	C9H9NO
		11	[4-hydroxyphenyl] acetonitrile	17.961	0.75	133.15	C8H7NO
		12	2-Hydroxy-3-methoxybenzaldehyde	19.085	0.33	152.15	C8H8O3
		13	Acetosyringone	19.479	0.21	196.2	C10H12O4
		15	levoglucosan	20.382	3.86	162.14	C6H10O5
		16	D-Glucose	20.697	2.92	180.16	C6H12O6
		17	1-Methoxy-1,2,3,4-tetrahydronaphthalene	21.05	0.44	162.23	C11H14O
		18	D-Fructose, 3-O-methyl	21.782	60.15	192.18	C7H14O6
		19	Arbutin	22.401	0.3	272,25	C12H16O7
		21	Methyl palmitate	25.217	0.21	270.5	C17H34O2
		22	Methyl alpha-D-glucopyranoside	25.413	0.29	194.18	C7H14O6
		23	l-[+]-Ascorbic acid 2,6-dihexadecanoate	25.682	0.6	652.9	C38H68O8
		24	Ethyl linolenate	28.528	0.65	306.5	C20H34O2
10	5	1	L-Thymidine	8.869	0.34	242.23	C10H14N2O5
		2	Undecane	9.645	1.02	156.31	C11H24
		3	Oleic acid	10.722	0.41	354.6	C21H42O2Si
		4	N-Methyl-L-proline	11.72	5.47	129.16	C6H11NO2
		5	Cyclotetrasiloxane	12.913	0.24	296.621	C8H24O4Si4
		6	Propanoic acid	13.269	0.69	162.14	C6H10O5
		8	1,3,5-Triazin-2[1H]-one	14.238	0.11	97.08	C3H3N3O
		9	1-[4-hydroxy-3-methylphenyl]ethanone	14.75	1.35	150.17	C9H10O2
		10	Cyclopentasiloxane	15.971	0.16	370.77	C10H30O5Si5
		11	Pyrrolidine	16.282	0.15	71.12	C4H9N
		12	Methyl alpha-D-glucopyranoside	17.229	11.82	194.18	C7H14O6
		13	1H-Indole, 2,3-dihydro-4-methyl	17.676	0.13	133.19	C9H11N
		14	4-Hydroxybenzyl cyanide	17.965	0.34	133.15	C8H7NO
		15	Arbutin	18.156	0.06	272,25	C12H16O7
		17	Acetosyringone	19.491	0.09	196.2	C10H12O4
		18	4-Methylpentanoic acid	19.683	0.42	116.16	C6H12O2
		20	levoglucosan	20.301	2.12	162.14	C6H10O5
		21	D-Glucose	20.653	0.59	180.16	C6H12O6

		22	D-Fructose, 1-O-methyl	21.228	68.53	192.18	C7H14O6
		23	Hexanoic acid	22.403	0.09	116.16	CH3[CH2]4COOH
		24	16-heptadecenal	23.945	0.29	252.4	C17H32O
		25	Methyl palmitate	25.231	1.14	270.5	C17H34O2
		26	l-[+]-Ascorbic acid 2,6-dihexadecanoate	25.687	0.58	652.9	C38H68O8
		27	9-Octadecenoic acid, methyl ester	27.893	1.63	296.4879	C19H36O2
		28	Phytol	28.062	0.4	296.5	C20H40O
		1	Propanenitrile, 3,3'-iminobis	6.345	1.32	123.16	C6H9N3
		2	L-Thymidine	8.874	0.18	242.23	C10H14N2O5
		3	Undecane	9.651	0.14	156.31	C11H24
		4	Octanoic acid	10.726	0.69	228.37	C14H28O2
		5	Cyclotetrasiloxane	10.859	0.78	296.621	C8H24O4Si4
		6	N-Methyl-L-proline	11.935	0.61	129.16	C6H11NO2
		7	Propanoic acid	13.277	0.49	162.14	C6H10O5
		8	trans-2-Octen-1-Ol	13.763	0.04	128.21	C8H16O
		9	hexahydro-1,3-diazocine-2[1H]-thione	14.057	0.73	144.24	C6H12N2S
		10	1-[4-hydroxy-3-methylphenyl]ethanone	14.753	1.17	150.17	C9H10O2
		11	Pyrrolidine	16.223	0.49	71.12	C4H9N
10	8	12	Sucrose	17.411	9.6	342.3	C12H22O11
		13	4-Hydroxybenzyl cyanide	17.687	0.21	133.15	C8H7NO
		14	[4-hydroxyphenyl] acetonitrile	17.968	0.76	133.15	C8H7NO
		15	levoglucosan	18.254	0.24	162.14	C6H10O5
		16	L-Pyroglutamic acid	18.813	6.36	129.11	C5H7NO3
		17	2-Hydroxy-3-methoxybenzaldehyde	19.099	1.56	152.15	C8H8O3
		18	Phenol, 4-methoxy-3-[methoxymethyl]	19.297	0.95	168.19	C9H12O3
		19	2-Phenylcyclopropanecarbonitrile	19.495	1.44	143.18	C10H9N
		21	Methyl alpha-D-glucopyranoside	20.394	5.48	194.18	C7H14O6
		22	alpha-D-Glucopyranoside,	20.716	2.15	342.3	C12H22O11
		23	Methyl alpha-D-mannopyranoside	21.5	53.7	194.18	C7H14O6
		24	Beta-D-allose	22.42	0.15	180.16	C6H12O6
		25	D-Glucose	22.702	0.19	180.16	C6H12O6
		28	Pentadecanal	23.947	0.07	226.4	C15H30O
		29	Methyl palmitate	25.233	0.2	270.5	C17H34O2
		30	l-[+]-Ascorbic acid 2,6-dihexadecanoate	25.692	0.5	652.9	C38H68O8
		31	Methyl oleate	27.896	0.08	296.5	C19H36O2
		32	Phytol	28.067	0.17	296.5	C20H40O
		33	Cetyl alcohol	28.551	0.08	242.44	C16H34O
		34	2-[[4-Methyl-1-oxo-2,3-dihydro-1H-inden-2-yl]methyl]benzoic acid	36.28	0.21	280.3	C18H16O3
		36	4',5,6,7-Tetramethoxyflavone	43.219	1.04	342.3	C19H18O6

Laser treatments [min]	*S. chinensis* Lines	Peak	Essential Oil Compounds	RT, min	Area, %	Molecular Weight	Molecular Formula
Control	1	1	4-Methyl-1,3-dioxane	10.71	0.29	102.13	C5H10O2
		2	N-Methyl-L-proline	11.636	14.46	129.16	C6H11NO2
		3	1-methylpyrrolidine-2-carboxylic acid	11.758	7.53	129.16	C6H11NO2
		4	[R]-Dimethyl 2-hydroxysuccinate	13.338	0.09	162.14	C6H10O5
		5	hexahydro-1,3-diazocine-2[1H]-thione	14.149	0.08	144.24	C6H12N2S
		6	2,6-Diethylcyclohexanone	14.211	0.06	154.25	C10H18O
		7	1-[4-hydroxy-3-methylphenyl]ethanone	14.734	0.63	150.17	C9H10O2
		8	Pyrrolidine	16.206	0.08	71.12	C4H9N
		9	Methyl alpha-D-glucopyranoside	17.34	3.9	194.18	C7H14O6
		10	2-Methoxyphenylacetonitrile	17.674	0.21	147.17	C9H9NO
		11	[4-hydroxyphenyl] acetonitrile	17.961	0.75	133.15	C8H7NO
		12	2-Hydroxy-3-methoxybenzaldehyde	19.085	0.33	152.15	C8H8O3
		13	Acetosyringone	19.479	0.21	196.2	C10H12O4
		15	levoglucosan	20.382	3.86	162.14	C6H10O5
		16	D-Glucose	20.697	2.92	180.16	C6H12O6
		17	1-Methoxy-1,2,3,4-tetrahydronaphthalene	21.05	0.44	162.23	C11H14O
		18	D-Fructose, 3-O-methyl	21.782	60.15	192.18	C7H14O6
		19	Arbutin	22.401	0.3	272,25	C12H16O7
		21	Methyl palmitate	25.217	0.21	270.5	C17H34O2
		22	Methyl alpha-D-glucopyranoside	25.413	0.29	194.18	C7H14O6
		23	l-[+]-Ascorbic acid 2,6-dihexadecanoate	25.682	0.6	652.9	C38H68O8
		24	Ethyl linolenate	28.528	0.65	306.5	C20H34O2
10	5	1	L-Thymidine	8.869	0.34	242.23	C10H14N2O5
		2	Undecane	9.645	1.02	156.31	C11H24
		3	Oleic acid	10.722	0.41	354.6	C21H42O2Si
		4	N-Methyl-L-proline	11.72	5.47	129.16	C6H11NO2
		5	Cyclotetrasiloxane	12.913	0.24	296.621	C8H24O4Si4
		6	Propanoic acid	13.269	0.69	162.14	C6H10O5
		8	1,3,5-Triazin-2[1H]-one	14.238	0.11	97.08	C3H3N3O
		9	1-[4-hydroxy-3-methylphenyl]ethanone	14.75	1.35	150.17	C9H10O2
		10	Cyclopentasiloxane	15.971	0.16	370.77	C10H30O5Si5
		11	Pyrrolidine	16.282	0.15	71.12	C4H9N
		12	Methyl alpha-D-glucopyranoside	17.229	11.82	194.18	C7H14O6
		13	1H-Indole, 2,3-dihydro-4-methyl	17.676	0.13	133.19	C9H11N
		14	4-Hydroxybenzyl cyanide	17.965	0.34	133.15	C8H7NO
		15	Arbutin	18.156	0.06	272,25	C12H16O7
		17	Acetosyringone	19.491	0.09	196.2	C10H12O4
		18	4-Methylpentanoic acid	19.683	0.42	116.16	C6H12O2
		20	levoglucosan	20.301	2.12	162.14	C6H10O5
		21	D-Glucose	20.653	0.59	180.16	C6H12O6
		22	D-Fructose, 1-O-methyl	21.228	68.53	192.18	C7H14O6
		23	Hexanoic acid	22.403	0.09	116.16	CH3[CH2]4COOH

		24	16-heptadecenal	23.945	0.29	252.4	C17H32O
		25	Methyl palmitate	25.231	1.14	270.5	C17H34O2
		26	l-[+]-Ascorbic acid 2,6-dihexadecanoate	25.687	0.58	652.9	C38H68O8
		27	9-Octadecenoic acid, methyl ester	27.893	1.63	296.4879	C19H36O2
		28	Phytol	28.062	0.4	296.5	C20H40O
10	8	1	Propanenitrile, 3,3'-iminobis	6.345	1.32	123.16	C6H9N3
		2	L-Thymidine	8.874	0.18	242.23	C10H14N2O5
		3	Undecane	9.651	0.14	156.31	C11H24
		4	Octanoic acid	10.726	0.69	228.37	C14H28O2
		5	Cyclotetrasiloxane	10.859	0.78	296.621	C8H24O4Si4
		6	N-Methyl-L-proline	11.935	0.61	129.16	C6H11NO2
		7	Propanoic acid	13.277	0.49	162.14	C6H10O5
		8	trans-2-Octen-1-Ol	13.763	0.04	128.21	C8H16O
		9	hexahydro-1,3-diazocine-2[1H]-thione	14.057	0.73	144.24	C6H12N2S
		10	1-[4-hydroxy-3-methylphenyl]ethanone	14.753	1.17	150.17	C9H10O2
		11	Pyrrolidine	16.223	0.49	71.12	C4H9N
		12	Sucrose	17.411	9.6	342.3	C12H22O11
		13	4-Hydroxybenzyl cyanide	17.687	0.21	133.15	C8H7NO
		14	[4-hydroxyphenyl] acetonitrile	17.968	0.76	133.15	C8H7NO
		15	levoglucosan	18.254	0.24	162.14	C6H10O5
		16	L-Pyroglutamic acid	18.813	6.36	129.11	C5H7NO3
		17	2-Hydroxy-3-methoxybenzaldehyde	19.099	1.56	152.15	C8H8O3
		18	Phenol, 4-methoxy-3-[methoxymethyl]	19.297	0.95	168.19	C9H12O3
		19	2-Phenylcyclopropanecarbonitrile	19.495	1.44	143.18	C10H9N
		21	Methyl alpha-D-glucopyranoside	20.394	5.48	194.18	C7H14O6
		22	alpha-D-Glucopyranoside,	20.716	2.15	342.3	C12H22O11
		23	Methyl alpha-D-mannopyranoside	21.5	53.7	194.18	C7H14O6
		24	Beta-D-allose	22.42	0.15	180.16	C6H12O6
		25	D-Glucose	22.702	0.19	180.16	C6H12O6
		28	Pentadecanal	23.947	0.07	226.4	C15H30O
		29	Methyl palmitate	25.233	0.2	270.5	C17H34O2
		30	l-[+]-Ascorbic acid 2,6-dihexadecanoate	25.692	0.5	652.9	C38H68O8
		31	Methyl oleate	27.896	0.08	296.5	C19H36O2
		32	Phytol	28.067	0.17	296.5	C20H40O
		33	Cetyl alcohol	28.551	0.08	242.44	C16H34O
		34	2-[[4-Methyl-1-oxo-2,3-dihydro-1H-inden-2-yl]methyl]benzoic acid	36.28	0.21	280.3	C18H16O3
		36	4',5,6,7-Tetramethoxyflavone	43.219	1.04	342.3	C19H18O6
